# Fully-Automated High-Throughput NMR System for Screening of Haploid Kernels of Maize (Corn) by Measurement of Oil Content

**DOI:** 10.1371/journal.pone.0159444

**Published:** 2016-07-25

**Authors:** Hongzhi Wang, Jin Liu, Xiaoping Xu, Qingming Huang, Shanshan Chen, Peiqiang Yang, Shaojiang Chen, Yiqiao Song

**Affiliations:** 1 Shanghai University of Medicine and Health Science, Shanghai, China; 2 Shanghai Key Laboratory of magnetic resonance, East China Normal University, Shanghai, China; 3 National Maize Improvement Centre, China Agricultural University, Beijing, China; 4 Schlumberger-Doll Research, Cambridge, Massachusetts, United States of America and Mass General Hospital, Charlestown, Massachusetts, United States of America; Institute of Genetics and Developmental Biology, Chinese Academy of Sciences, CHINA

## Abstract

One of the modern crop breeding techniques uses doubled haploid plants that contain an identical pair of chromosomes in order to accelerate the breeding process. Rapid haploid identification method is critical for large-scale selections of double haploids. The conventional methods based on the color of the endosperm and embryo seeds are slow, manual and prone to error. On the other hand, there exists a significant difference between diploid and haploid seeds generated by high oil inducer, which makes it possible to use oil content to identify the haploid. This paper describes a fully-automated high-throughput NMR screening system for maize haploid kernel identification. The system is comprised of a sampler unit to select a single kernel to feed for measurement of NMR and weight, and a kernel sorter to distribute the kernel according to the measurement result. Tests of the system show a consistent accuracy of 94% with an average screening time of 4 seconds per kernel. Field test result is described and the directions for future improvement are discussed.

## Introduction

Recently NMR has been widely used in material characterization, such as rubber crosslink density testing [1–2], food quality testing [3–5], rock core analysis [3,6–8], porosity of building materials such as cement and wood [3,9,10]. The oil content of seeds determines the value of crops, e.g. [11–13]. The measurement of oil and moisture content in crop seeds is important for the production, processing, storage and sale of oilseeds [14,15].

One of the modern crop breeding technologies uses doubled haploid (DH) plants which contain a pair of identical chromosomes [16,17]. For instance, cultivars purity can reach 98% by using traditional pedigree method only after 8 generations of selection, but the purity could achieve 100% in two generations using the DH technology [17].

Haploid refers to the gametophyte chromosome number. The haploid breeding is a technology that uses anther culture or gynogenesis to produce haploid, and then obtains new stable genotypes through natural or artificial doubling. The percentage of spontaneous haploid is found to be 1% in nature and thus not an effective breeding method. Doubled haploid can be produced by in vitro or in vivo induction methods with a significantly higher rate of 8~15% [18–23]. The high oil content inducer (such as the CAUHOI line [18]) allows the potential identification of haploids based on kernel oil content detectable by NMR.

Several approaches to identify haploid seeds have been used, such as field identification, conventional cytogenetic, morphological method, radioactive method, herbicide method, molecular marker method, genetic marker method [18]. For example, one haploid identification method is making use of ‘Navajo’ kernel trait: kernels with a pigmented endosperm and a non-pigmented embryo are selected as haploids. However, the expression of anthocyanin gene (and thus the color) can be affected by donor genotype and environment. Meanwhile, visual fatigue of the operators will reduce the accuracy of the screening. In comparison, the NMR approach is based on the oil content measured directly by NMR and can be scaled up to a high-throughput system to satisfy the practical high-volume selection process [24–27], such as screening millions of seeds in a two-week period at a continuous rate of screening about 1 kernel per second.

NMR has been used for nondestructive oil analysis of seeds [28] and materials. Kotyk et al. used a 1.5T whole-body MRI scanner with a 1-m diameter bore to analyze the oil content of 2592 corn kernels that are arranged on a cubic sample holder at a rate of 1 kernel per second [29]. Based on continuous wave-free precession (CWFP) sequence, Alberto et al. have used a superconducting magnet with a 2.1-T magnetic field and a 30 cm bore to analyze more than 20,000 seeds per hour, that is 5 kernels per second [30]. However, the actual rate limiting step of these systems is the manual handling and weighing of each kernel [29].weight of individual kernels was not reported[30].

In this paper, we describe a fully-automated, high-throughput NMR system for DH technology. The system automatically weighs each kernel and measures its oil content individually. It then sorts the kernel according to its oil content ratio (oil content divided by total weight). The current system has achieved 20,000 seeds per day at a rate of 1 kernel every 4 seconds. We will describe its design principle, major components, and examples of its operation and results.

## Materials and Methods

### Measurement of oil and water content by NMR

#### Measurement of oil and water content in corn

**Fig 1:**
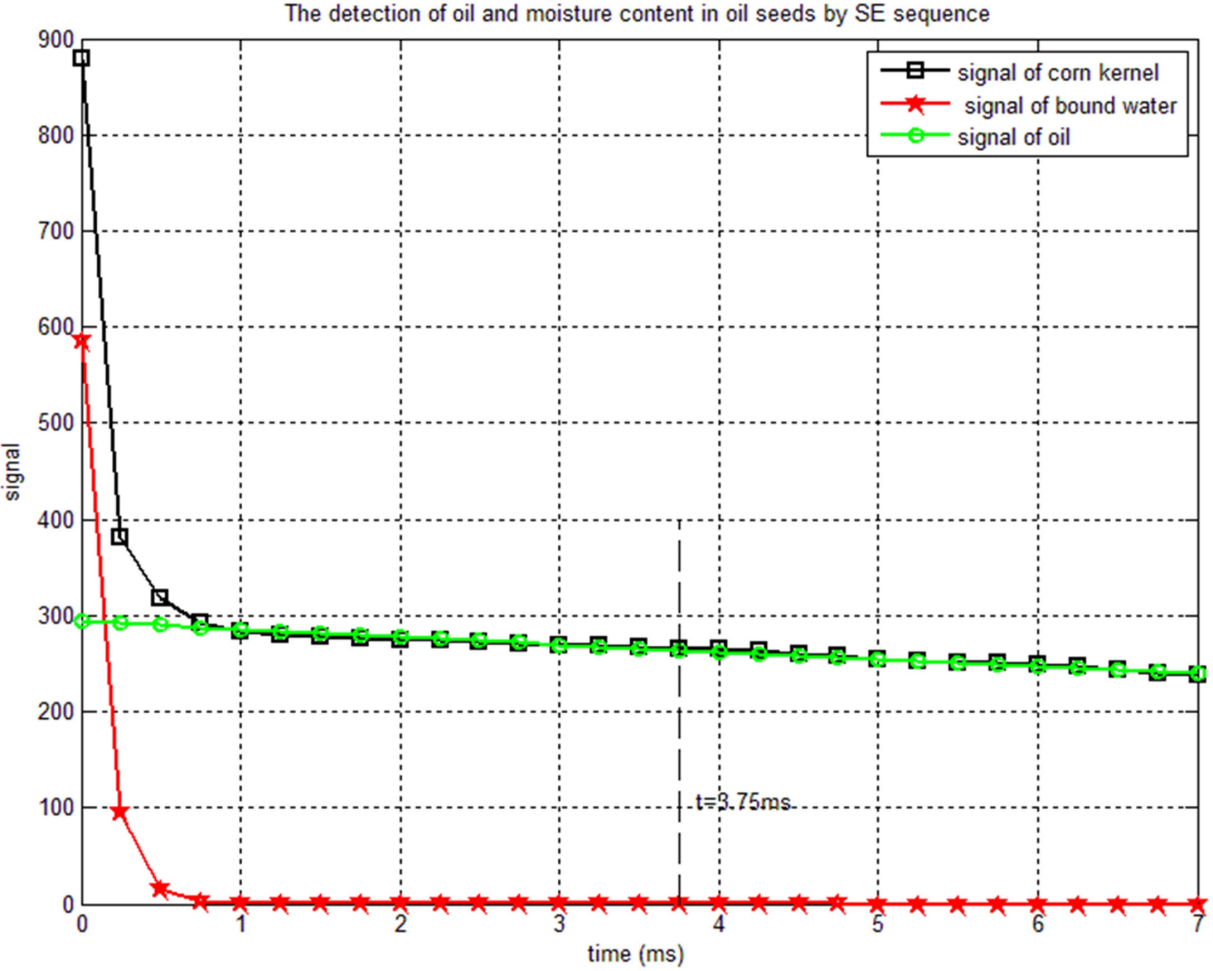
Plot of the echo amplitude (open squares) as a function of half echo time. The green line is the signal of bound water and the red line that of oil. Since the bound water decays quickly, thus the signal at TE = 7.5 ms is only contributed from the oil.

The main fluid components in a corn kernel are water and oil. It is well-known that the signal for oil and water exhibits significantly different T_2_ [31]. We use spin-echo sequence in order to distinguish the oil and water signal and an example is shown in Fig 1.

The spin echo signal from corn kernels (S(t)) follows a bi-exponential function:

$$S\left(\text{t}\right)=S_{\text{o}}\text{exp}\left(-\frac{\text{t}}{{\text{T}}_{2\text{o}}}\right)+S_{\text{w}}\text{exp}\left(-\frac{\text{t}}{{\text{T}}_{2\text{w}}}\right)$$ (1)

Where S_o_ and **S**_w_ are respectively the protons density of oil and water in the seed. T_2o_ and T_2w_ are the transverse relaxation time of oil and water, respectively. At t = 0, the signal amplitude ***S***(0) = ***S***_o_+***S***_w_ is the sum of the hydrogen of water and oil. ***S***(0) is also measured by the FID signal right after the first 90 degree pulse. The Larmor frequency used was 22.06 MHz, the 90/180 degree pulses are 4/8 us long and detection bandwidth is 100KHz. Recycle time is 500 ms and two or four signals were accumulated according to the oil content of corn. The amplitude at zero time is obtained by FID.

A double exponential fitting is shown as the red and green lines in Fig 1. The green line stands for the fast relaxation component (bound water), and the red line is the slower component (oil). T_2o_ and T_2w_ in seeds are clearly different. The water in seeds after drying can be considered as bound water when the moisture content is lower than 15%, and T_2w_ is short, typically shorter than 1 ms. On the other hand, the oil T_2o_ is long, typically longer than 80ms. Our data shows T_2o_ = 100 ms and T_2w_ = 0.75 ms. As a result, for an echo time TE = 7.5 ms, the water contribution to the signal is essentially zero (exp(-7.5/0.75) = 0.00005) and the echo signal only contain oil contribution which can be expressed as:

$$S(TE)=S_{o}exp\left(-\frac{TE}{T_{2o}}\right)∼S_{o}$$

Thus the water content S_w_ = *S(0)*-S_o_.

#### Calibration of oil and water content

In order to obtain quantitative water and oil content (A) in seeds, we compare the measurement NMR amplitude (S, in unit of volt) to the weight (mg) of water and oil of seed. In our system, a small background signal (b) can be observed, so that the oil and water content can be obtained by

**Fig 2:**
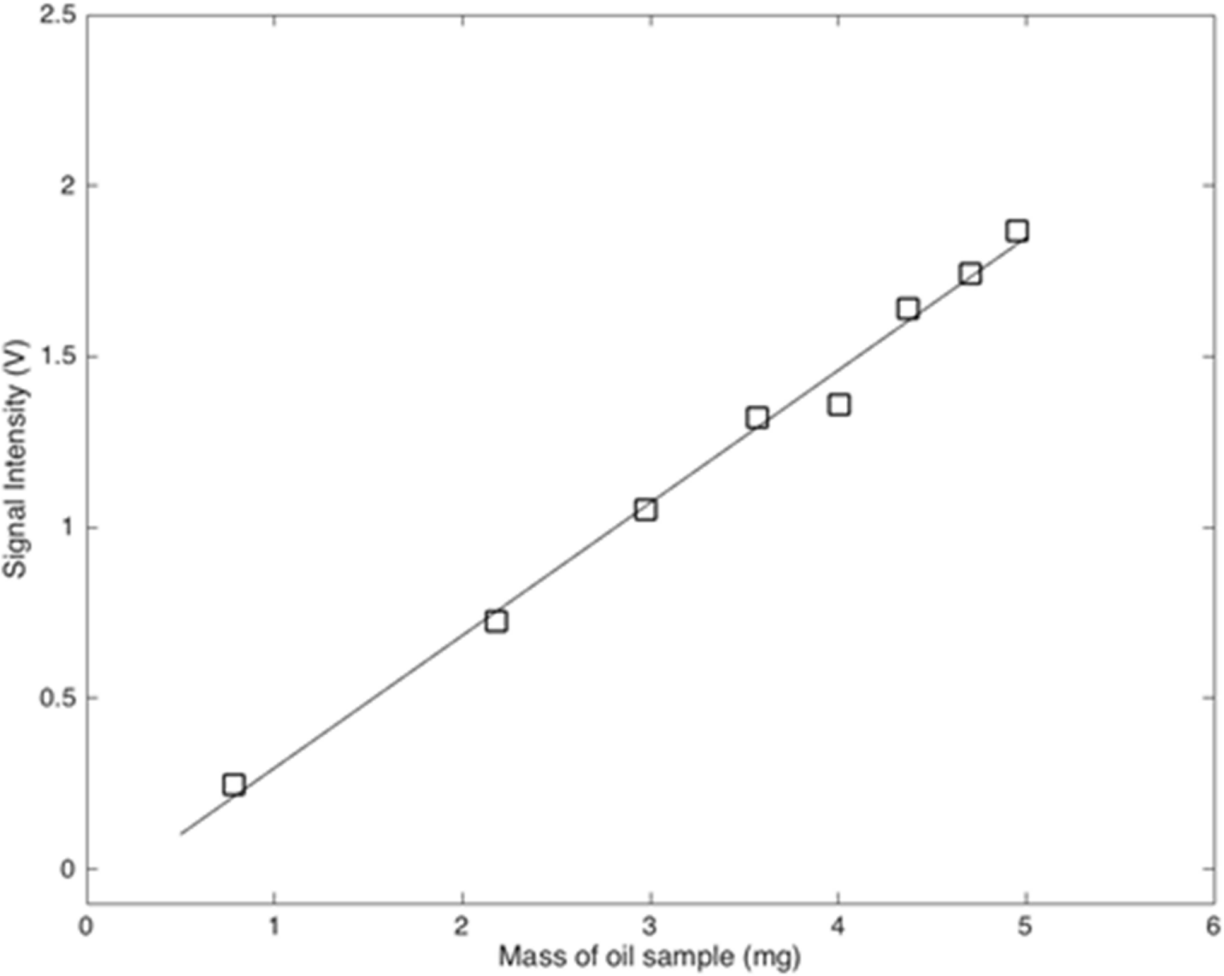
Plot of the NMR signal vs the mass of oil samples for system calibration.

A=kS+b (2)

k is the calibration constant. Experimental data in Fig 2 shows a linear relationship between S and A and the calibration constant is k = 361.7 mg/V, obtained by a least-square fit. This calibration is repeated every 24 hours in order to maintain the up-to-date calibration during screening processes.

Finally, the oil (or water) content ratio (OCR or WCR) is acquired by calculating A/m, where m is the mass of the sample obtained by the weighing sensor. It is important to note that the measurement condition should be kept the same at each measurement, such as NMR pulse sequence parameters, and sample and instrument temperatures.

### Fully automatic high-throughput NMR system

The objective of this system is to measure the weight and oil content of each individual kernel and to sort it according to its oil content ratio. The system is comprised of three electronic-mechanical subsystems: 1) Single kernel feeder before the NMR unit, it selects a single kernel, weighs it and feeds it into the NMR unit; 2) NMR unit with a sample holder to perform NMR; 3) Kernel sorter distributes the kernels based on the result.

#### Single kernel feeder

**Fig 3:**
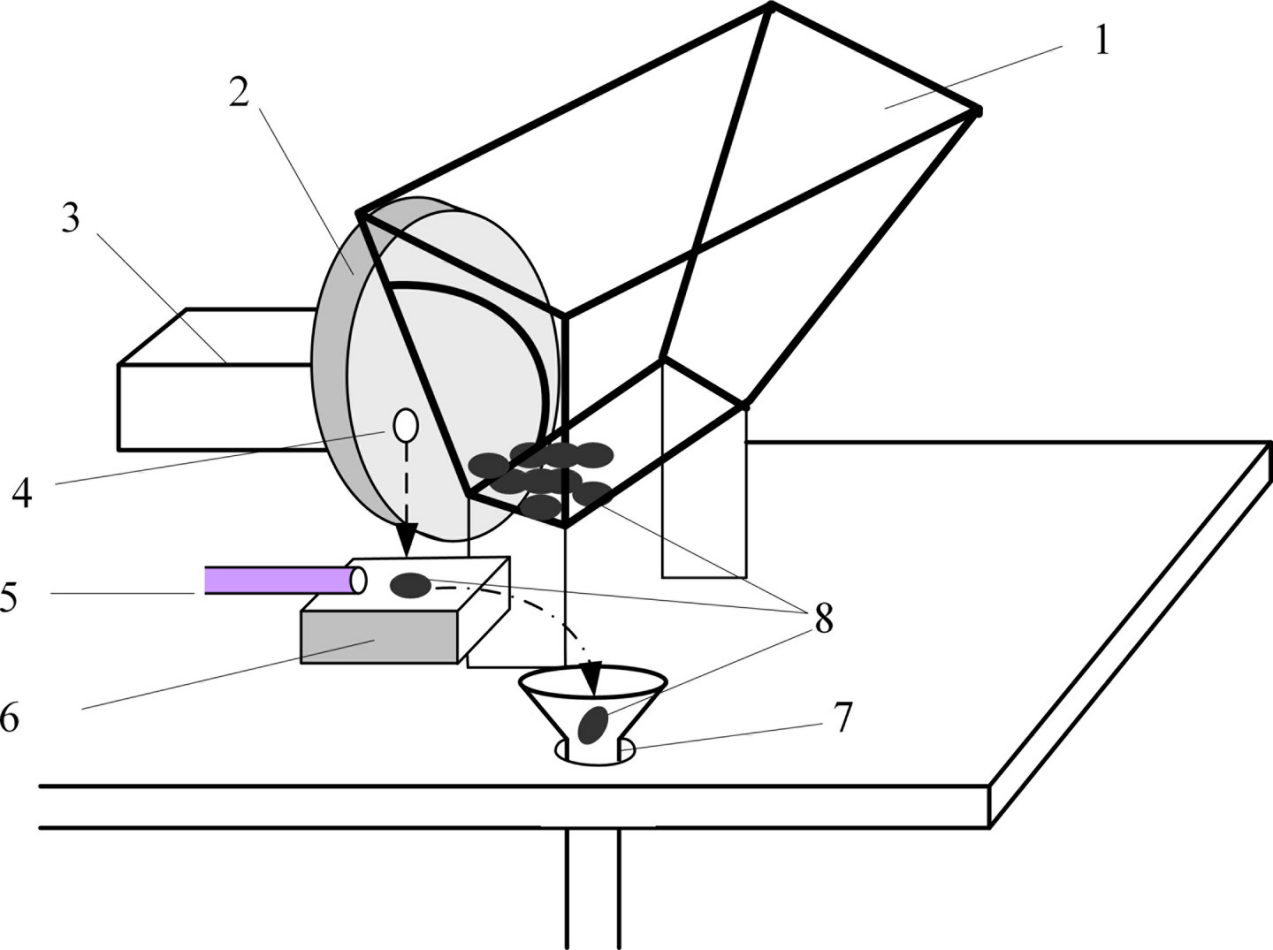
Schematic diagram of the single kernel feeder. 1: Sample hopper; 2: Picker unit that sucks in a single kernel; 3: Stepping motor unit; 4: Pit; 5: Sample blowing pipe; 6: Weighing sensor; 7: Sample pipeline; 8: Kernels.

All major components of the feeder are shown in Fig 3:

1. Sample hopper, 25 cm long, 12 cm wide and 30 cm tall. Kernels enough for 24-hours operation may be loaded.
2. Picker unit that sucks in a single kernel.
3. Stepping motor unit that rotates the picker.
4. Pit, an indentation on the picker unit that is connected to a vacuum generator.
5. Sample blowing pipe connected to an air compressor.
6. Weighing sensor.
7. Sample pipeline that blows the kernel into the NMR sample holder.

The micro-controller controlled stepping motor unit (3) periodically rotates the picker unit (2) to the outlet of the hopper unit (1) that contains the corn kernels. The vacuum generator applies a negative pressure to the vacuum chamber in the picker unit in order to suck a single kernel into the picker unit at the position of the pit (4). The pit is a small indentation on the surface of picker unit large enough to hold one kernel and connected with the vacuum chamber. The picker unit then rotates to move the selected kernel to the weighing sensor (6) and drops the kernel by releasing the vacuum. And then the seed falls onto the weighing sensor. After a 1-s wait time for stability, the weight of the kernel is measured and the data is sent to the control software. Then, a puff of positive pressure air from sample blowing pipe (5) will push the seed into the sample pipeline (7) and the seeds will drop into the NMR sample holder for NMR measurement.

#### NMR sample holder

**Fig 4:**
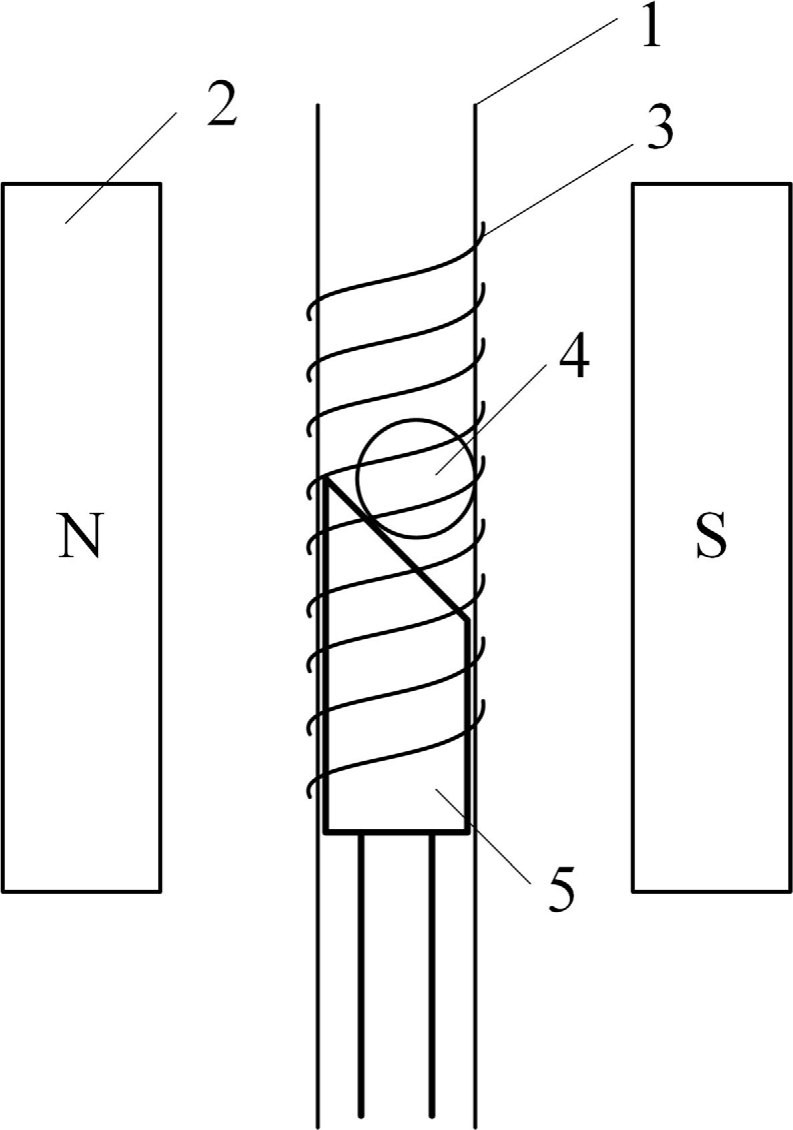
Schematic diagram of the NMR sample holder. 1: Sample pipeline; 2: Magnet; 3: RF coil; 4: Kernel; 5: Sample plug.

As show in Fig 4, NMR sample holder consists of an RF coil (3), sample pipeline (1) and a sample plug (5). After entering into the sample pipeline, the kernel (4) will be stopped by the sample plug and positioned in the center of the magnet. Then, NMR measurement will be performed to obtain the oil content of the kernel. When the NMR measurement is complete, the sample plug moves downwards to lower the kernel into the kernel sorter. Once the sample is past to the sorter, the sample plug will be raised to return to the center of the magnet (2) for the next measurement.

#### Kernel sorter

**Fig 5:**
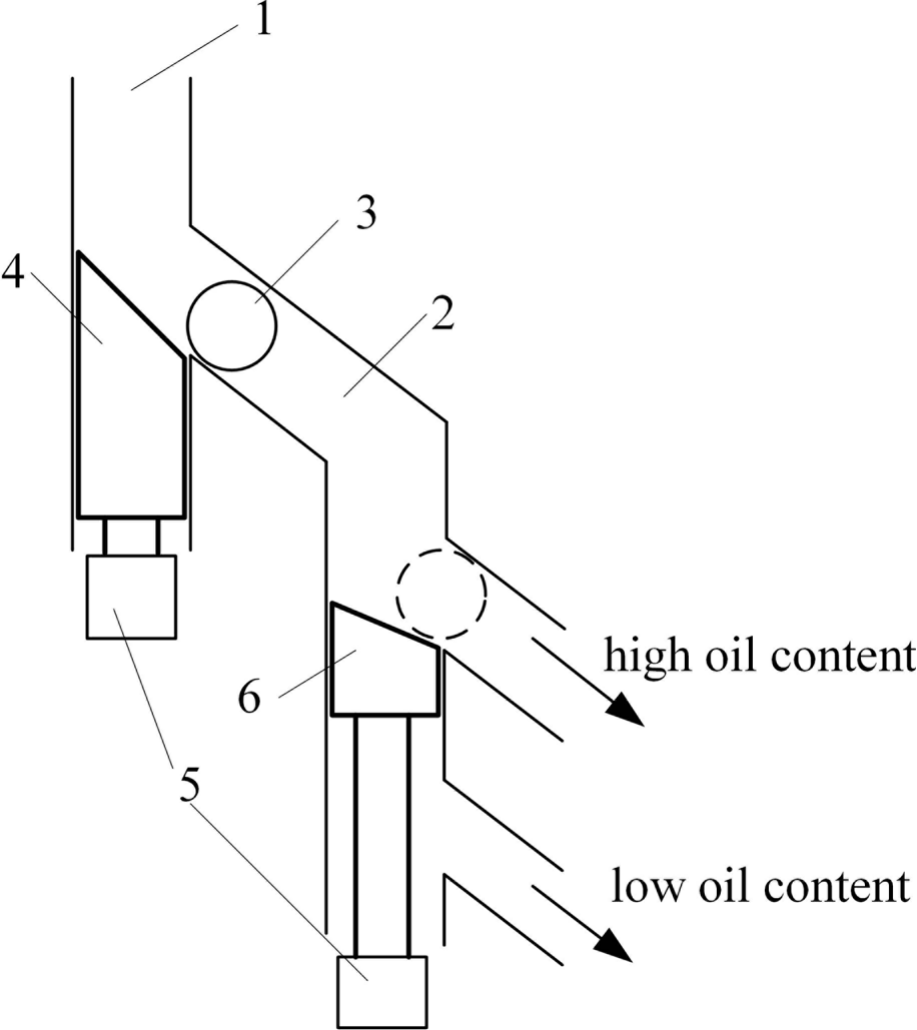
Schematic diagram of the kernel sorter. 1: Sample pipeline; 2: Sort pipeline; 3: Kernel; 4: Sample plug; 5: Cylinder; 6: Sorting plug.

As shown in Fig 5, the kernel sorter consists of sort pipeline, sorting plug and high/low oil content pipelines. After the kernel (3) enters into the sort pipeline (2) from the sample pipeline (1), the sorting plug (6) will position itself according the measured oil content ratio to match the outlet pipeline (high or low oil content ratio) so that the kernel could slide off the corresponding pipeline by its own weight. The sorting plug and sample plug are driven by cylinder (5).

#### System control software

**Fig 6:**
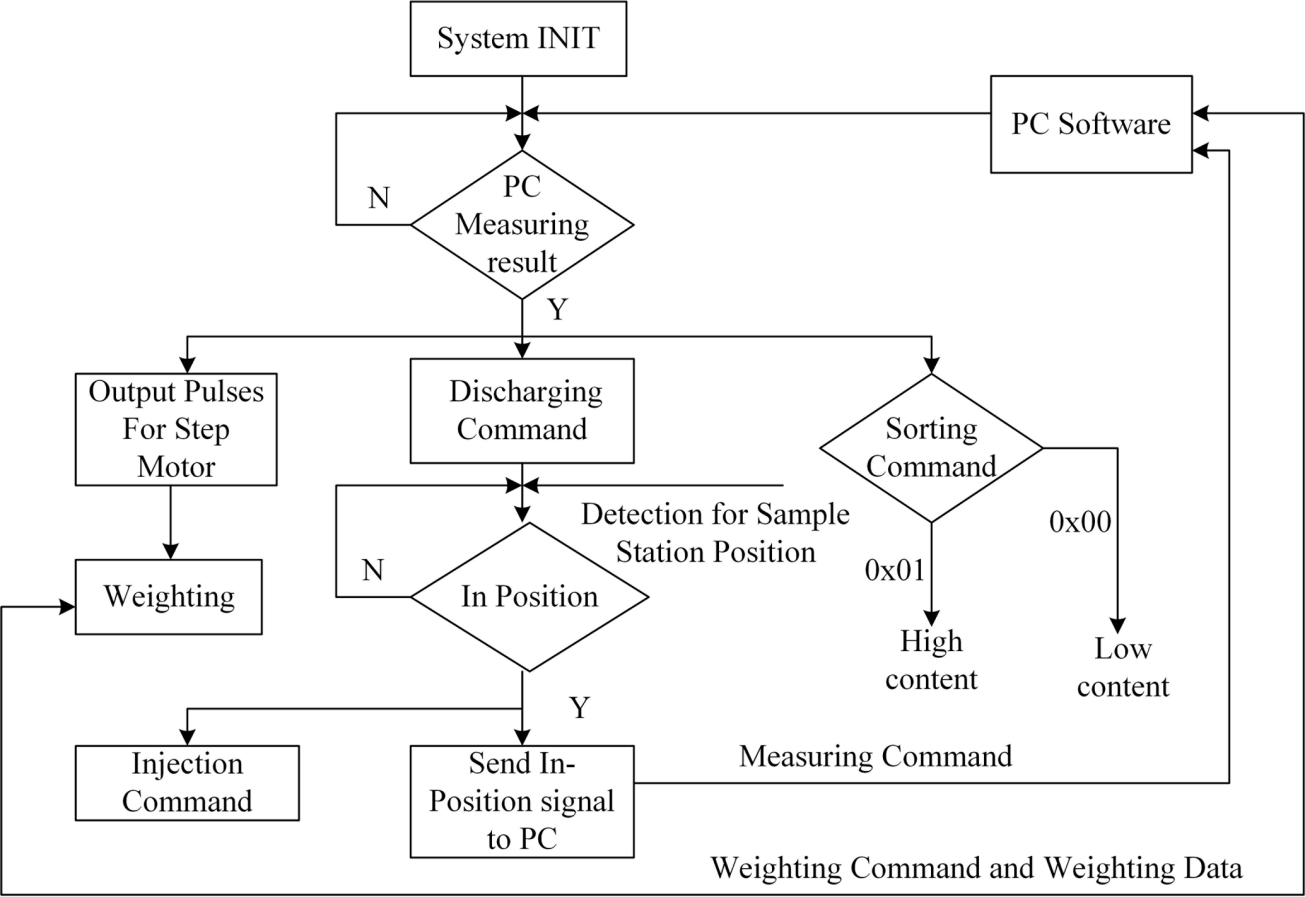
The block diagram of the software for the automatic measurement and sample handling.

The operation of the system hardware is fully controlled by the computer software integrated with the measurements. Fig 6 shows the flowchart of the automation system control software. There are three major steps of the operation:

1. Sampling: using picker to suck in a kernel and drop it onto the weighing sensor; the weight of the kernel is taken;
2. NMR: compressed air will push the selected kernel into the sample pipeline and NMR sample holder; NMR measurement is performed;
3. Sorting: dependent on the oil content and the sample weight, the kernel is sorted into high or low oil content ratio bins.

To ensure the high success rate of the single kernel sampling, at each step of the operation, the system software checks the passage of the kernel via several sensors. For example, there is a magnetic switch to determine if the sample plug is inside the NMR magnet and this sensor is read by the system software. Also, after each weighing, the weighing sensor is reset after the kernel is loaded into the NMR sample pipeline.

## Results and Discussions

**Fig 7:**
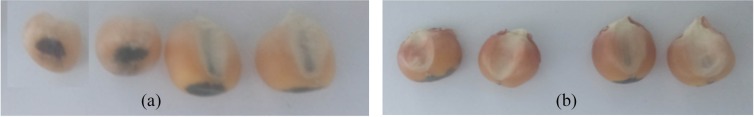
The photographs of the haploid and diploid of Zheng58 and Yu87-1. (a) Zheng58, the left 2 kernels are haploid, and the right 2 kernels are diploid; (b) Yu87-1, left 2 kernels are haploid and the right 2 kernels are diploid.

To meet the demand of large-scale DH breeding of maize, National Maize Improvement Centre of China Agricultural University (CAU, Beijing) has applied this system to screen maize haploid kernels. Fig 7 shows the haploid and diploid kernels of zheng58 and Yu87-1 which were induced by CAUHOI. CAUHOI is an inducer lines with high oil content which was developed by CAU[18].

### Optimal NMR measurement time for a single corn

**Fig 8:**
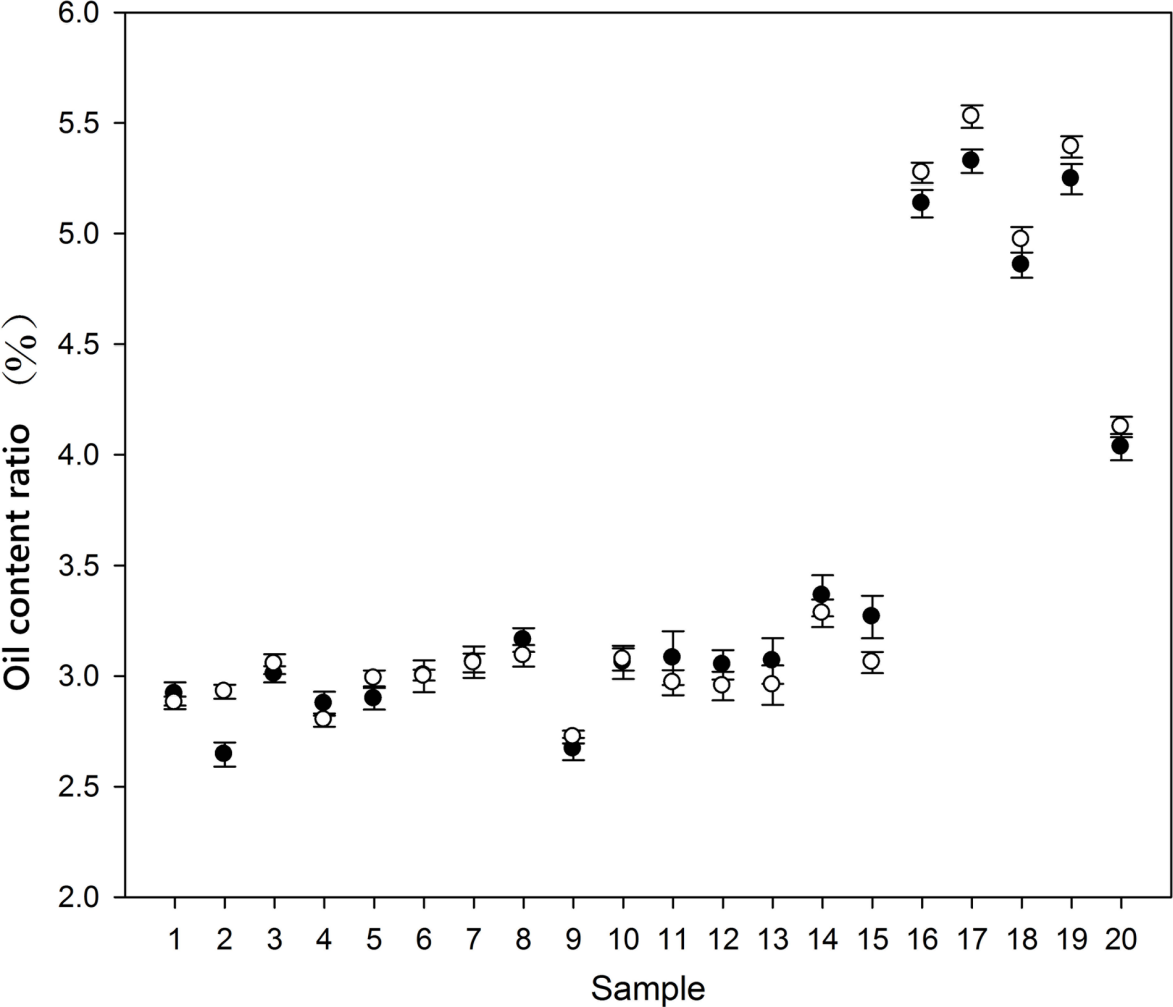
A plot of the oil content ratio measured by using two measurement times 1 sec (filled circles) and 2 sec (open circles) showing no significant differences.

In order to accelerate the speed of screening without affecting the accuracy of haploid identification, twenty F1 seeds (15 seeds of XY335 and 5 seeds of Zheng58/CAUHOI) randomly selected were measured for 10 times with measurement time of 1 and 2 s, and the oil contents obtained by the two different measurement times were compared. The result (Table 1 and Fig 8) shows no significant differences between the two measurement times. And linear regression fit indicates that oil content is significantly correlated (r = 0.994) with two measurements. Based on the above results, from sample feeding to discharge, the average speed of screening using this system will be 4 seconds per kernel.

**Table 1:** Test of statistical significance of the accuracy obtained under different acquisition times. Note: d*f* is degree of freedom; Sig. (2 tailed): the two tailed value probability value; *y*1 and *y*2: oil content ratio at measurement time of 1 and 2 s, respectively. The original data for this analysis is shown in Fig 8.

	parameter					*t* value	d*f*	Sig. (2 tailed)
	mean	STD	Standard error	99% confidence interval				
				lower	Upper			
Difference	-0.02	0.122	0.027	-0.098	0.059	-0.71	9	0.487

### Stability test

To validate the stability of the automatic screening system, the same kernel Zheng58/CAUHOI of 0.42g was tested 50 times using the NMR measurement time of 1 second. We found that the maximum oil content ratio: 7.2%, the minimum: 6.5%, the average: 6.7%, and the standard deviation: 0.7%. For this line, the oil content ratio of the diploid maize is more than twice that of the haploid, so the system stability can fully meet the demand of haploid screening.

### Test success rate of the single kernel selection

**Fig 9:**
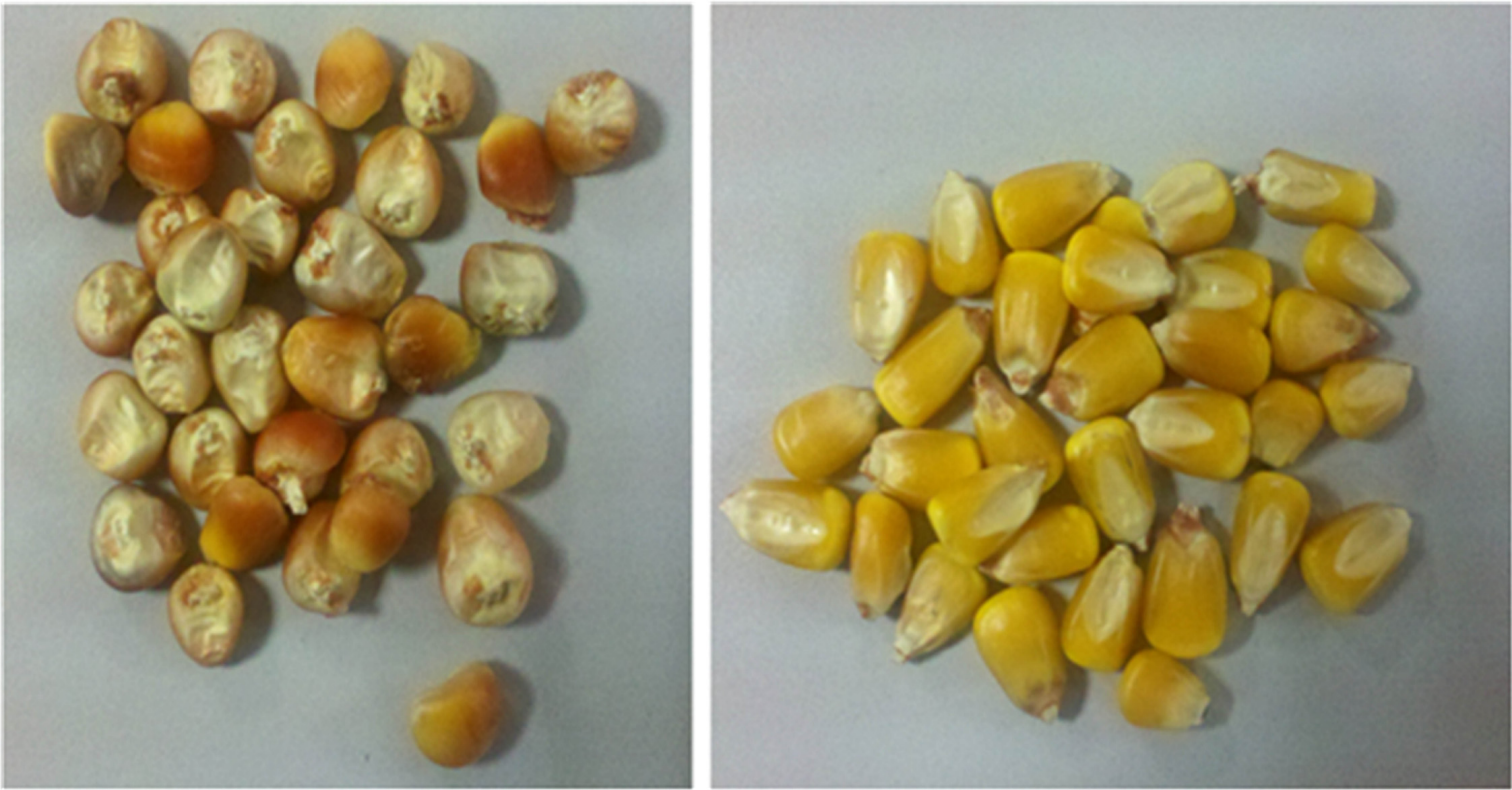
Kernels of different shapes. Left: round shape corns; right: flat shape corns.

We continuously tested the round and the flat corns (Fig 9) separately for ten hours in order to examine the success rate of the single kernel selection. In the 10-hour test of 9490 measurements of the round corns, the pit fails 672 times to load the single kernel, and thus the success rate is 92%. For the flat corns, the success rate was found 90%, slightly lower than that for the round corns. For other round seeds such as peanut and soybean, we found even higher success rate up to 98%.

### Test of soybeans

This high-throughput system was also used to measure oil and water content of two types of soybean seeds, one is larger (0.35–0.6g) and the other one smaller (0.12–0.3g). Fifty of the two types each were measured for a total of 13462 times. The success rate of the single kernel feeder in selecting a single seed is 97.4%. This result of better success rate is expected because soybean has much more regular in shape and size than corn kernels.

These two types of soybeans, named Feng1 and Feng2 produced by Northeast Institute of geography and agricultural ecology, Chinese Academy of Sciences, are special breeds for their high oil content. For Feng1, the measured mean OCR is 17.5% with a STD 2.0%. For Feng2, the measured mean OCR is 20.4% with a STD of 1.0%. These results show that Feng2 is not only higher in oil content, but also with much reduced variation in oil content among the seeds.

### Field test of maize kernels

We carried out a field test of this screening system for ZD958 maize kernels induced by high oil inducer material CHOI3 [27]. The endosperm and embryo of diploid are color-labeled, but for haploid, only the endosperm is color-labeled. Because of being influenced by the genetic background of maternal germplasm and environment, the color label on the embryo of diploid is not very obvious from the photographs alone. In addition, the size, shape and quality between haploid and diploid are very similar, so the manual selection based on visual inspection in fact can be challenging.

**Fig 10:**
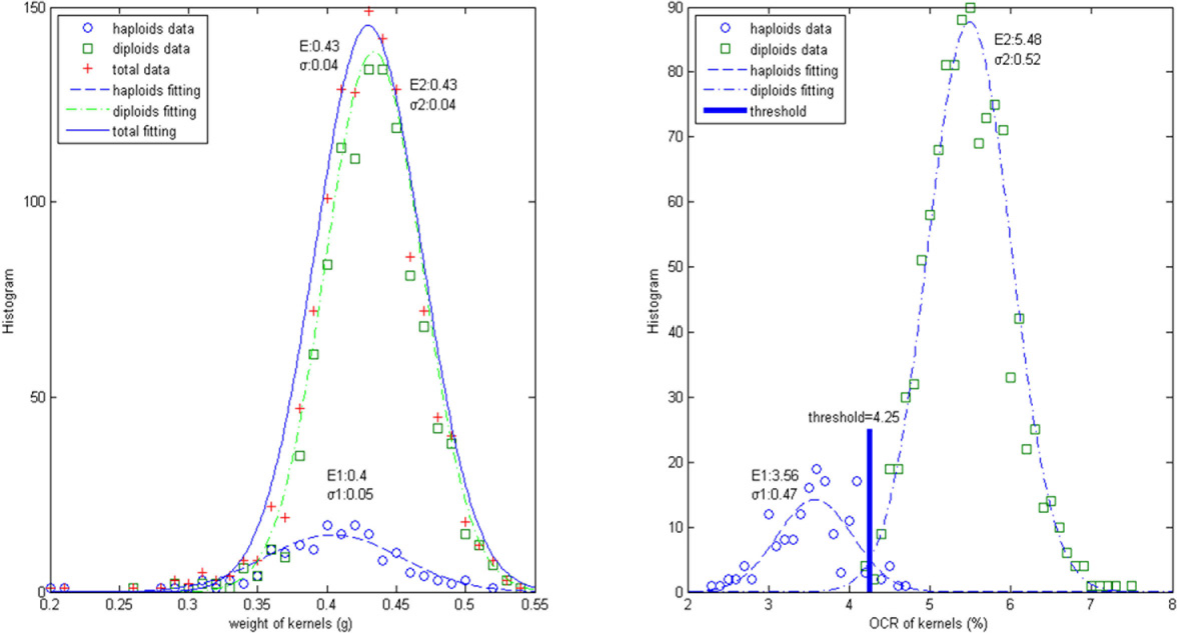
**Left panel:** The distribution of ZD958 kernels weight (haploids, diploids and total kernels are shown as circles, squares and pluses, respectively) and their fitting distributions (haploids, diploids and total kernels are shown as a dash line, a dot-dash line and a solid line respectively). **Right panel:** The OCR distribution of ZA958 kernels (haploids and diploids are shown as circles and squares respectively) and their fitting distributions (haploids and diploids are shown as a dash line and a dot-dash line respectively). It shows that an OCR threshold of 4.25% can well distinguish diploids from haploids.

We completed 1260 measurements in about 1.5 hours using our full automatic NMR system. The kernels were first sorted by the manual visual inspection to select diploids from haploids. Then, the kernels were fed into the NMR system for automated analysis. The experimental results of weight and oil content ratio of the kernels are shown in Fig 10. The OCR result shows a clear separation of haploid and diploid kernels.

The distribution of weight and OCR appears to be consistent with a Gaussian form and the fitting is shown in Fig 10A and 10B respectively. The means and STD of the weight distribution of haploid and diploid kernels are 0.40(E1), 0.049(σ1) and 0.43(E2), 0.035(σ2), separately. The weight difference between diploids and haploids (E1-E2 = 0.03) is less than the width of the distributions ((σ1+σ2)/2 = 0.04), thus there are no significance differences in weight between haploid and diploid seeds and they can't be identified by weight alone.

Fig 10 shows the statistics distribution of the oil content ratio of the 1260 kernels with a peak value of OCR at 3.6% and STD of 0.47 for haploid, OCR at 5.5% and STD at 0.52 for diploid, respectively. The deference (E2-E1 = 1.9%) is clearly larger than the width of the distributions ((σ1+σ2)/2 = 0.5%), thus the haploid /diploid shows a significance OCR difference and the threshold at 4.25% can clearly distinguish the haploid. Using this threshold, the false negative probability, which is the possibility of diploid mis-selected to be haploid is 0.83%, and the false positive probability, which is the possibility of haploid mis-selected to be diploid is 7.27%, the total false screening probability is 8.2%. In breeding technology, both the false positive and negative probabilities must be controlled strictly.

The addition of the weight sensor is critical in obtaining OCR as the weight of the seeds varies significantly. Based on the oil content alone, 3.4% of diploid would be selected as haploid which accounts for 23% of the haploids, resulting in a much worse (by a factor of 4) selection compared to the use of OCR.

This method has been applied at CAU to examine several crosses with the high oil induction line CHOI3 [18]. The comparison of the NMR system with the conventional manual selection method by color labels is shown in Table 2 with an average success rate of 94%.

**Table 2:** Accuracy of the screening system. The results are from the first filial generation (F1) seeds of six crosses that were screened by the NMR system.

Crosses or hybrids	Total number of seeds	Haploids by NMR	True Haploids	Accuracy (%)
ZD958	3742	321	308	95.95
XY335	3807	315	299	94.94
Zhongnongda622	3269	266	237	89.1
B73/Zheng58	3591	303	279	92.08
Xu178/Dan3130	3316	287	274	95.47
Chang7-2/Ji853	3520	310	290	93.55
Total	21245	1802	1687	94

The high-throughput system described has achieved very high speed of oil content (in unit of g of oil) measurement[29]. However, it relies on manual handling of individual seeds for weight and loading the MRI sample container. As such, the overall speed is still impressive. However, the equipment used is a clinical MRI system with 1.5-T superconducting magnet which is highly expensive in acquisition, installation, operation and maintenance. Our NMR system uses a permanent magnet at a lower field and thus is much less expensive with low maintenance cost. The use of a much smaller RF coil tightly enclosing the sample makes the detection highly efficient and high SNR. As a result, the much lower-cost instrument of ours provides highly accurate data of oil content. The single-kernel feeder and the integrated weighing sensor allow the system to automatically measure OCR without any manual intervention and to operate continuously. The field tests of our systems have demonstrated the robust operation of our system.

Our current single kernel selection system shows a fail rate of up to 10% that need to be improved. The vacuum system may cause noticeable vibration and noise. We are currently updating the mechanical design of the single-kernel selection system to further reduce its fail rate and vibration coupling to the NMR system. Furthermore, NMR unit needs to be improved to accelerate the overall throughput of the system with a goal of one kernel per second.

## Conclusion

This paper describes in details a fully-automated high-throughput NMR screening system for haploid maize selection and there need not any pre-treatment or post treatment to the kernels. We show that the combination of automated NMR and weight measurement is a key to obtain OCR and demonstrate the successful operation of the system in our laboratory and in field tests. Such fully automated system could accelerate corn breeding programs and reduce cost. It may also be useful for other agriculture and economically important seeds, such as oil seeds to obtain OCR and work specific to these fields is needed to further validate such application.
